# Genomic and transcriptomic insights into Achromobacter–Sphingobium co-colonization within polycyclic aromatic hydrocarbon-exposed bacterial communities

**DOI:** 10.1099/mic.0.001712

**Published:** 2026-05-21

**Authors:** Mana Sato, Robert A Kanaly, Jiro F Mori

**Affiliations:** 1 Graduate School of Nanobioscience, Yokohama City University, Yokohama, Japan

**Keywords:** *Achromobacter*, aromatic hydrocarbon biodegradation, microbial interaction, *Sphingobium*, transcriptomics

## Abstract

Efficient and complete biodegradation of polycyclic aromatic hydrocarbons (PAHs), which are persistent and genotoxic petroleum hydrocarbon pollutants, is often considered to require the cooperative activities of multiple bacterial groups, and bacterial (meta)genomic investigations of PAH-exposed ecosystems have contributed to elucidating such interactions. In this study, two bacterial isolates representing dominant genera within a PAH-grown soil bacterial consortium, *Achromobacter xylosoxidans* strain KK8 and *Sphingobium barthaii* strain KK22, were utilized as model organisms to investigate the relationship between these bacterial genera during PAH biodegradation. Strain KK8 has previously been characterized as incapable of biodegrading PAHs; thus, *Achromobacter* in the consortium appears to grow under metabolic dependence on PAH biodegradation products (i.e. salicylic acid) provided by the pioneer PAH-degrading *Sphingobium*. This metabolic relationship was evidenced through complete genome sequencing and functional gene analysis of strain KK8 conducted in the present study. To further elucidate potential interactions between *Achromobacter* and *Sphingobium*, cell-free filtrate-exchange experiments were performed using these isolates, revealing that strain KK8 exhibited a significantly shortened growth lag phase in the presence of the filtrate of strain KK22. Subsequent transcriptomic profiling of strain KK8 indicated that exposure to the *Sphingobium* filtrate up-regulated functional genes likely associated with *Achromobacter* colonization, including genes involved in biofilm formation (*pga* genes) or cell division (*fts* genes). Enhanced biofilm formation of strain KK8 in response to strain KK22 filtrate was additionally evidenced by biofilm assays. Taken together, these results suggest that the high abundance of *Achromobacter* within the consortium may be stimulated by *Sphingobium* when they are present together, potentially via extracellular signalling molecule(s). As the co-occurrence of *Achromobacter* and *Sphingobium* has been repeatedly documented in PAH-degrading bacterial communities, elucidating the mechanisms underlying their specific interspecies co-colonization during PAH biodegradation shall be valuable for the future biotechnological applications utilizing these bacteria.

**Impact Statement**

Strains of *Achromobacter* and *Sphingobium*, both isolated from a diesel fuel-grown soil bacterial consortium and with fully sequenced genomes, were utilized as model organisms to investigate the mechanisms underlying the co-occurrence of these two genera that have repeatedly been documented. The metabolic dependencies of *Achromobacter* on *Sphingobium*, as well as interspecies interactions mediated by signalling mechanisms that trigger *Achromobacter* colonization, were first elucidated through bioassays and transcriptomic analyses of the *Achromobacter* strain using the cell-free filtrate of *Sphingobium*. The insights obtained in this study shed light on the ‘black box’ of microbial interactions within petroleum-impacted ecosystems and may be valuable for future bioremediation applications.

## Data Summary

The genome sequence of strain KK8 is available in the National Center for Biotechnology Information (NCBI) GenBank under the accession number CM135178 and the IMG/MER database under accession number 8120043210. The raw genome sequence data and raw RNA-seq data are available in the NCBI Sequence Read Archive under accession numbers PRJNA1345530 and PRJNA1345567.

## Introduction

Bacteria capable of biodegrading petroleum hydrocarbon pollutants such as the highly hydrophobic, persistent and (geno)toxic polycyclic aromatic hydrocarbons (PAHs) have attracted considerable research interest in the context of bioremediation and in understanding the environmental fates of these pollutants [1,3]. Aerobic biodegradation of PAHs is initialized by the introduction of two oxygen atoms into the aromatic rings by aromatic ring-hydroxylating dioxygenases (ARHDs) [4,5], which is followed by cleavage of the oxidized rings by aromatic ring-cleaving dioxygenases (ARCDs) [6,7], which often results in the formation of a variety of biotransformation products with relatively higher bioavailability [8,9]. It has thus been considered that the co-occurrence and cooperative activities of the ‘pioneer’ PAH degraders that possess ARHD and ARCD enzymes with other microbial groups that consume PAH biodegradation products are likely required for the efficient and complete biodegradation of complex PAH pollutants [10,12]. However, the specific cooperative interactions among microbial players in petroleum-exposed environments remain largely unexplored and are therefore often referred to as a ‘black box’ [13,14].

In a previous study investigating petroleum hydrocarbon biodegradation by multispecies bacterial communities, metagenomic profiling of a soil bacterial consortium enriched with diesel fuel revealed that the consortium consisted of more than ten bacterial genera after multiple transfers over 15 years [11]. According to that study, when the consortium was cultured separately with the PAHs naphthalene or phenanthrene as the sole sources of carbon and energy, a pioneering PAH-degrading genus, *Sphingobium*, and a co-existing genus, *Achromobacter*, were identified as highly abundant within the consortium. Biodegradation tests of bacterial isolates originating from the consortium revealed that the *Sphingobium* isolate (*Sphingobium barthaii* strain KK22) was capable of biotransforming a variety of PAHs [15,19], and this ability has been genomically characterized through complete genome sequencing (GenBank accession numbers CP060035–CP060038; IMG Genome ID 2883162986) [9,20, 21], whereas the *Achromobacter* isolate (strain KK8) was able to grow on single-ring aromatic hydrocarbons but lacked the ability to utilize PAHs as growth substrates [11]. These results suggested that *Achromobacter* was enriched in the consortium through metabolic dependencies by utilizing PAH biodegradation products produced from *Sphingobium*. In fact, enrichment of PAH-degrading *Sphingobium* species together with co-colonizing *Achromobacter* species has been repeatedly reported in PAH-degrading soil bacterial communities in previous studies [22,24]. Collectively, these findings indicate that members of the genus *Achromobacter* may have evolved, and broadly conserved, the ability to specifically co-colonize with other PAH-degrading bacteria such as *Sphingobium* and dominate bacterial communities by outcompeting other bacterial groups potentially occupying similar ecological niches.

The current study aims to elucidate the potential specific relationship between *Achromobacter* and *Sphingobium* species that have repeatedly been found to co-occur in PAH-exposed environments, with particular emphasis on the mechanisms that cause the high abundance of the non-PAH-degrading *Achromobacter* in the same communities with the PAH-degrading *Sphingobium*, utilizing bacterial isolates of these genera derived from the same consortium as model micro-organisms. To achieve this goal, complete genome sequencing of strain KK8 was conducted, followed by transcriptomic analysis of its response to the cell-free filtrate of strain KK22. The results of these analyses revealed the potential mechanisms by which *Achromobacter* interacts with *Sphingobium* and advantageously co-colonizes with it in PAH-degrading bacterial communities.

## Methods

### Culture conditions of *Achromobacter xylosoxidans* strain KK8 and *S. barthaii* strain KK22

*A. xylosoxidans* strain KK8 and *S. barthaii* strain KK22 were isolated from a diesel fuel-grown soil bacterial consortium by colony separation in previous studies, along with other strains [25,28]; strain KK8 was purified as a colony on nutrient agar [11], while strain KK22 was isolated on Noble agar (Becton, Dickinson Biosciences, San Jose, CA) with crystalline phenanthrene [29]. These bacterial isolates were cultivated in 25 ml of Stanier’s basal medium (SBM) in 100 ml glass conical flasks supplemented with either 50 mg l^−1^ salicylic acid (>99% purity; Wako Chemical, Osaka, Japan) or 1 mM glucose as the sole carbon source and incubated at 30 °C with rotary shaking at 120 r.p.m. in the dark.

### Genome sequencing of strain KK8

Genomic DNA of strain KK8 was extracted using the NucleoBond high-molecular-weight DNA Kit (Macherey-Nagel, Düren, Germany) from bacterial cells grown overnight in Luria-Bertani (LB) liquid medium and subsequently purified using DNA Clean Beads (MGI Tech, Shenzhen, China) [30,31]. SMRTbell template libraries were prepared with the SMRTbell Express Template Prep Kit 3.0 (PacBio, Menlo Park, CA, USA) using DNA sheared with a g-TUBE (~10–20 kbp; Covaris, Woburn, MA, USA), and sequencing was performed on the Revio platform at Bioengineering Lab. Co., Ltd. (Sagamihara, Japan) with the Revio polymerase kit (PacBio). After adapter trimming and quality filtering of raw reads using SMRT Link (PacBio; ver. 13.0.0.207600) and Filtlong (ver. 0.2.1) [32], HiFi reads longer than 1,000 bp were subjected to *de novo* assembly using Flye (ver. 2.9.3-b1797) [33]. The completeness and chromosome circularity of the assembled genome were assessed using CheckM (ver. 1.2.2) [34] and Bandage (ver. 0.8.1), a visualizing software for evaluating *de novo* genome assemblies [35]. Gene annotation was performed using the National Center for Biotechnology Information Prokaryotic Genome Annotation Pipeline (ver. 6.8) [36] and the Integrated Microbial Genomes (IMG) Annotation Pipeline (ver. 5.2.1) [37]. The average nucleic acid identity (ANI) between the genome of strain KK8 and those of reference bacterial strains was determined using pairwise ANI analysis on IMG. All software was used with default settings unless otherwise specified.

### Filtrate-exchange experiment between strains KK8 and KK22 cultures

To investigate the effects of bacterial filtrates on the growth of strains KK8 and KK22, filtrate-exchange experiments were conducted. Strains KK8 and KK22 were cultured for 3 days in SBM supplemented with 1 mM glucose, and each culture was then filtered through a 0.2 µm PTFE syringe filter (Merck, Darmstadt, Germany) to obtain cell-free filtrates. The filtrates obtained were mixed with fresh SBM at a 2 : 3 vol ratio (10 ml of filtrate and 15 ml of fresh SBM) in 100 ml glass conical flasks, and cells of strains KK8 and KK22 were inoculated into the respective filtrate-containing media, resulting in four experimental conditions: strain KK8 exposed either to the strain KK8 filtrate or the strain KK22 filtrate and strain KK22 exposed either to the strain KK8 filtrate or the strain KK22 filtrate. In all cases, 1 mM glucose was supplied as the carbon source. Additionally, cultures of strains KK8 and KK22 in 25 ml of fresh SBM without the filtrate and supplemented with 1 mM glucose were also prepared as controls. Differences in bacterial growth behaviour under these six conditions were initially pre-screened by measuring OD_600_. After this pre-screening, for detailed and continuous monitoring of the growth of strain KK8 in response to the strain KK22 filtrate, strain KK8 cultures exposed to the strain KK22 filtrate [KK8(KK22)] with or without 1 mM glucose, as well as control strain KK8 cultures without filtrate with 1 mM glucose, were prepared in 10 ml glass tubes, and OD_600_ was measured hourly using Taitec OD monitoring equipment (OD-Monitor A and S; Taitec Co., Ltd., Saitama, Japan).

### Transcriptomic analysis of strain KK8 in response to strain KK22 filtrate

Strain KK8 cells pre-grown on glucose for 3 days were harvested by centrifugation at 16,000 ***g*** for 5 min, resuspended in fresh SBM to an OD_600_ of 0.10 and further incubated with glucose either in the presence of strain KK22 filtrate [KK8(KK22); 2 : 3 vol ratio] or without it (control KK8), in triplicate for each condition. After 3 h of incubation, total RNA was extracted from each culture using the NucleoSpin RNA Kit (Macherey-Nagel), and rRNA was removed with the Ribo-Zero Plus rRNA Depletion Kit (Illumina, San Diego, CA, USA) [38]. Strand-specific cDNA libraries were prepared using the NEBNext Ultra II Directional RNA Library Prep Kit (New England Biolabs, Beverly, MA, USA) and sequenced on the Illumina NovaSeq X Plus platform (Illumina; 2×150 bp paired-end). After adapter trimming and quality filtering of raw reads using Fastp (ver. 0.23.4) [39], high-quality reads obtained were mapped to the assembled genome of strain KK8 using Bowtie2 (ver. 2.5.1) [40]. featureCounts (ver. 2.0.6) [41] was used to count the reads of each coding sequence, and differentially expressed genes (DEGs) between KK8(KK22) and control KK8 cultures were identified using the R-based tool edgeR package (ver. 4.2.1) [42] based on a log_2_ fold change (log_2_FC) and false discovery rate (FDR) thresholds. EnhancedVolcano package (ver. 1.22.0) in R was used to generate a volcano plot of DEGs.

### Biofilm formation assay

Biofilm formation in strain KK8 cultures was quantified using the crystal violet biofilm assay [38,43]. Strain KK8 cells pre-grown on glucose for 3 days were inoculated into 2 ml of either fresh SBM mixed with strain KK22 filtrate (2 : 3 vol ratio) or fresh SBM alone in a 10 ml glass tube containing 1 mM glucose. After static incubation in the dark at 30 °C, the cultures were discarded, and the tubes were gently rinsed three times with distilled water. Biofilms adhered to the tube walls were stained with 0.1% crystal violet (Becton Dickinson, Sparks, MD, USA). After removing excess stain by rinsing with distilled water, the retained crystal violet was eluted with 2 ml of ethanol and quantified by measuring absorbance at 570 nm using a 96-well plate reader. All assays were performed in triplicate, and statistical significance was evaluated using Welch’s t-test (*P*<0.05, *P*<0.01).

## Results

### Genomic characteristics of strain KK8

**Fig. 1.**
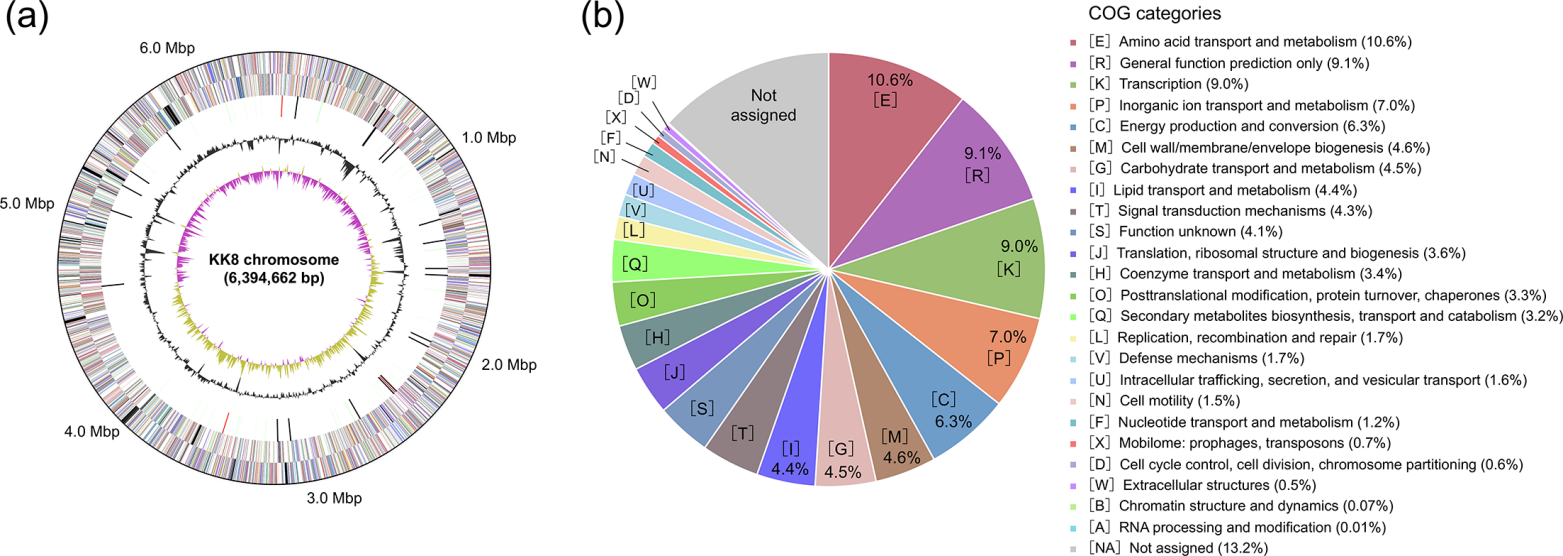
Complete genome sequence and functional gene annotation of *A. xylosoxidans* strain KK8. (**a**) Circular map of the strain KK8 chromosome. From outermost to innermost rings: predicted genes on the forward and reverse strands [coloured by the COG functional categories shown in panel (**b**)], RNA genes (tRNAs, green; rRNAs, red; other RNAs, black), GC content (grey) and GC skew (yellow and purple). (**b**) Relative abundance of predicted functional genes in the strain KK8 genome according to COG classification.

The complete genome of strain KK8, obtained through *de novo* assembly of PacBio long-read sequence data (Table S1, available in the online Supplementary Material), exhibited 100% completeness as assessed by CheckM and consisted of a single circular chromosome with a size of 6,394,662 bp (Fig. 1), with its circularity confirmed by Bandage (Fig. S1). The strain KK8 chromosome carries 5,764 CDSs, 10 rRNA genes and 68 tRNA genes, according to the IMG annotation pipeline. These CDSs were functionally categorized under Clusters of Orthologous Groups (COG) classification, revealing that the most abundant category was ‘[E] Amino acid transport and metabolism’ (12.22% of total CDSs), followed by ‘[R] General function prediction only’ (10.48%), ‘[K] Transcription’ (10.31%) and ‘[P] Inorganic ion transport and metabolism’ (8.03%). Taxonomic analysis revealed that strain KK8 was closely related to *A. xylosoxidans* strains SOLR10 (GenBank accession number CP025774) and NBRC 15126^T^ (CP006958), showing 99.10% and 99.01% ANI, respectively (Table S2), thus placing this strain within the *A. xylosoxidans* clade.

### Predictions of functional genes involved in aromatic hydrocarbon biodegradation in strain KK8

**Fig. 2.**
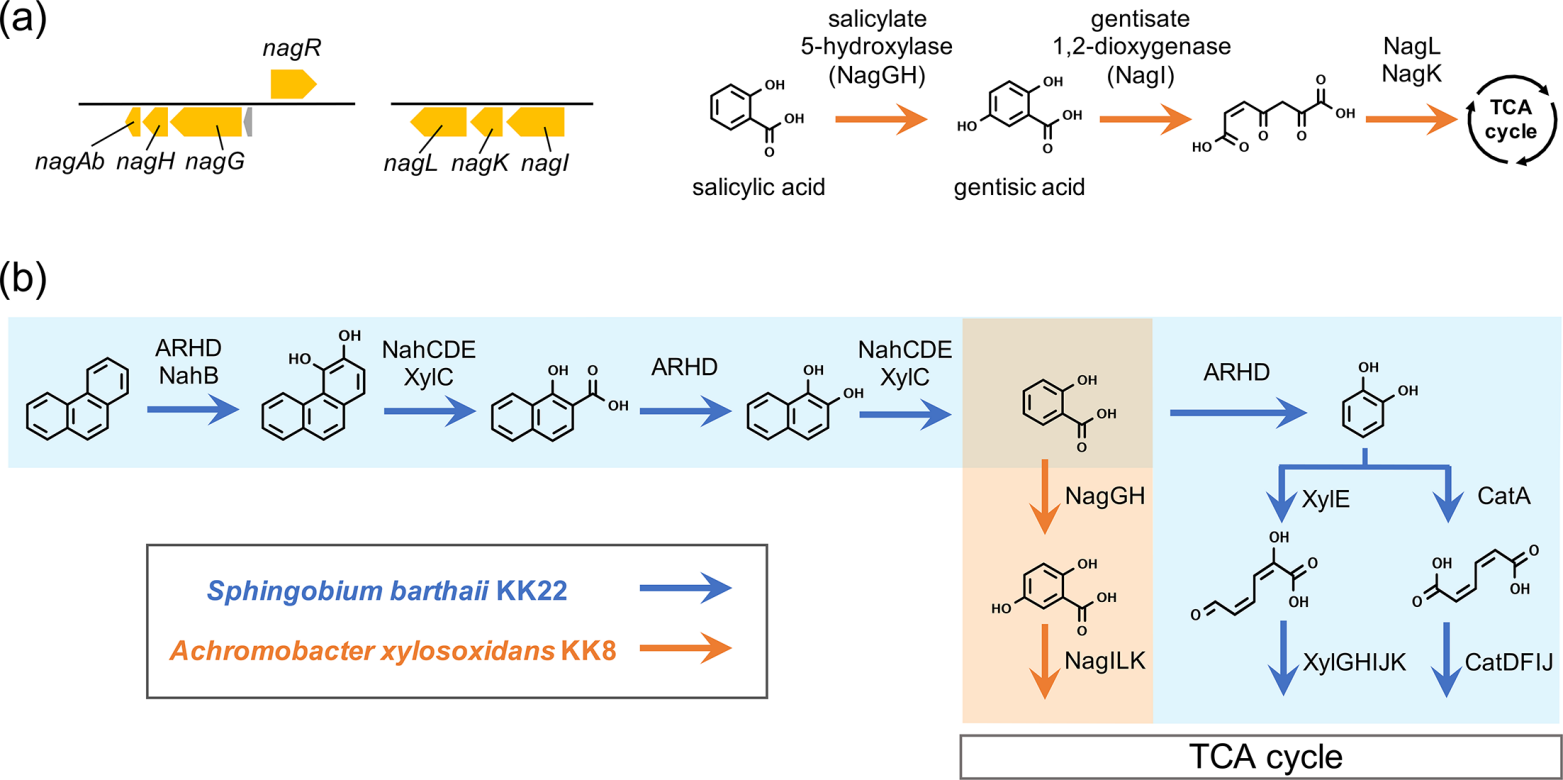
(**a**) Predicted functional gene clusters responsible for salicylic acid biotransformation in *A. xylosoxidans* strain KK8. (**b**) Proposed phenanthrene biodegradation pathway based on genomic information from *S. barthaii* strain KK22 and *A. xylosoxidans* strain KK8. Coloured arrows indicate the enzymes responsible for each biotransformation step, encoded by strain KK22 (blue) and strain KK8 (orange).

Strain KK8 was confirmed to grow on 50 mg l^−1^ salicylic acid as the sole carbon source, demonstrating its capability to biodegrade and utilize salicylic acid as a growth substrate. Functional genes involved in aromatic hydrocarbon biodegradation in strain KK8 were identified across the whole genome based on gene annotation pipeline results and comparative analyses with the genomes of other bacterial strains [44,45]. The genome of strain KK8 was found to contain putative homologous genes encoding Nag enzymes responsible for the biotransformation of salicylic acid (Fig. 2; Table S3); *nagG/nagH*, encoding salicylate 5-hydroxylase large/small subunits (EC 1.14.13.172), and *nagAb*, encoding the ferredoxin component, which together catalyse the conversion of salicylic acid to gentisic acid [46]; *nagR*, encoding a transcriptional regulator; *nagI*, encoding gentisate 1,2-dioxygenase (EC 1.13.11.4), responsible for the aromatic ring cleavage of gentisic acid; and *nagL* and *nagK*, encoding maleypyruvate isomerase (EC 5.2.1.4) and fumarylpyruvate hydrolase (EC 3.7.1.20), respectively, which catalyse the downstream degradation of ring-opened products [47]. These *nag* genes were also identified in reference genomes of *Achromobacter* in public databases (*A. xylosoxidans*, *Achromobacter denitrificans*, *Achromobacter insolitus*, *Achromobacter spanius* and *Achromobacter seleniivolatilans*; Table S4) and are highly conserved in the genomes of the plant-associated bacteria *A. xylosoxidans* strain SOLR10 and *A. insolitus* AB2 (GenBank accession number CP022199), both of which have been characterized as capable of biodegrading salicylic acid via gentisic acid [48], showing 83.5–100% amino acid sequence identity to the corresponding proteins in strain KK8 (Table S3). Putative homologues of other known functional genes involved in upstream PAH biodegradation were not identified in the genome of strain KK8, consistent with previous findings that strain KK8 was unable to grow on phenanthrene or naphthalene as the sole carbon source [11]. Conversely, strain KK22 has been characterized as possessing functional genes encoding enzymes responsible for PAH biotransformation, including several types of ARHDs, as well as Nah, Xyl and Cat enzymes (Fig. 2), which enable this strain to utilize PAHs as growth substrates [9,20].

### Shortened growth lag phase of strain KK8 upon exposure to strain KK22 filtrate

**Fig. 3.**
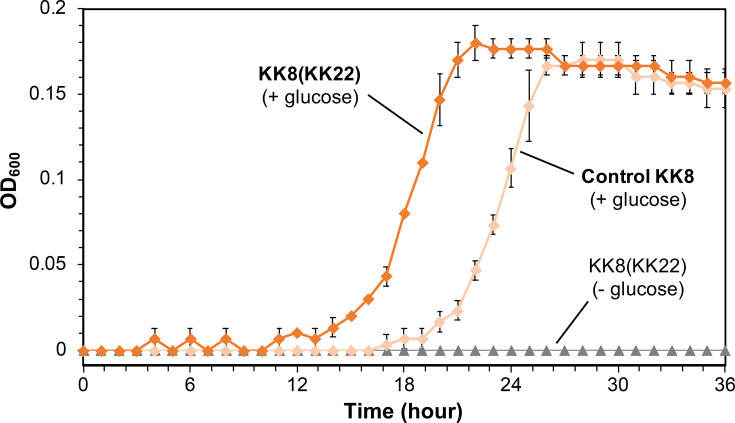
Growth curves of strain KK8 (diamond symbols) incubated with the filtrate of strain KK22 [KK8(KK22), orange] or without the filtrate of strain KK22 (control KK8, light orange), both supplied with 1 mM glucose. The strain KK8 cultures which were supplied with the filtrate of strain KK22 but lacking glucose are shown by grey triangles. Error bars represent sd (*n*=3).

Through pre-screening for the effects of the cell-free filtrates of strains KK8 and KK22 on the growth of each strain on glucose, it was found that strain KK8 cultures that were exposed to the strain KK22 filtrate exhibited more rapid growth when compared to the growth of strain KK8 cultures that were either (1) exposed to strain KK8 filtrate or (2) grown without any filtrate. Because no apparent difference in growth was observed when strain KK8 cells were exposed to strain KK8 filtrate or grown without filtrate, it was also concluded that self-inhibitory effects caused by strain KK8 metabolites were not observed. Additionally, no significant differences in growth were observed among strain KK22 cultures under all conditions tested, suggesting that the strain KK22 filtrate specifically affected the growth of strain KK8. Detailed growth monitoring of strain KK8 revealed that cultures supplied with the strain KK22 filtrate [KK8(KK22)] exhibited a shortened growth lag phase when compared to KK8 cultures that were incubated without filtrate (controls) that corresponded to an ~5 h reduction in the lag phase (Fig. 3). Strain KK8 cultures supplied with the strain KK22 filtrate but without glucose showed no growth after 36 h of incubation (Fig. 3), indicating that compounds present in the strain KK22 filtrate were not utilized as additional growth substrates by strain KK8.

### Transcriptional response of strain KK8 to the strain KK22 filtrate

**Fig. 4.**
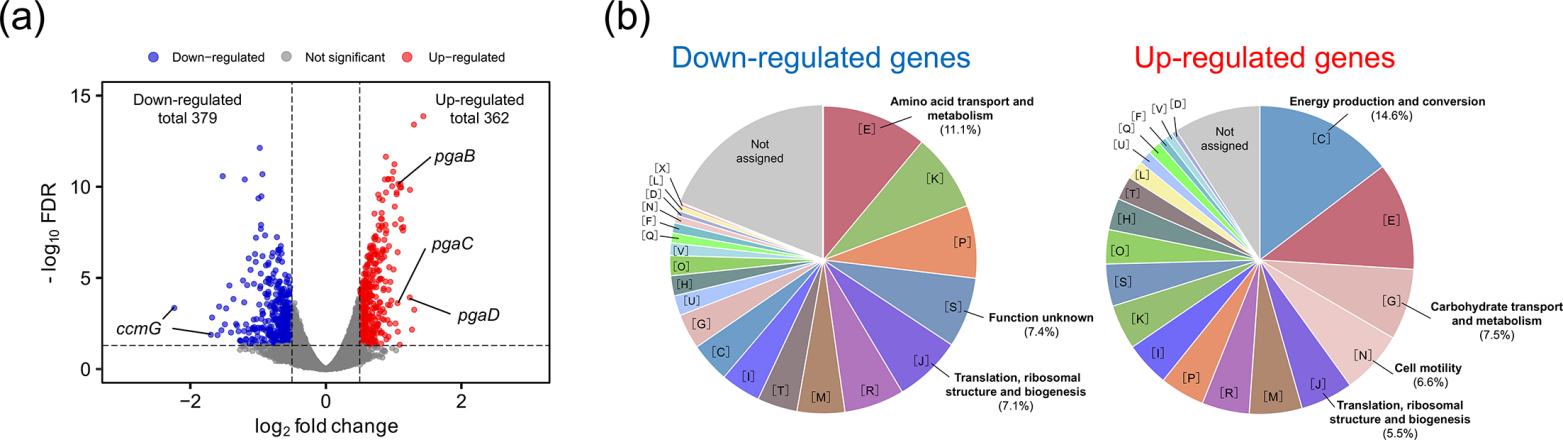
Transcriptomic profiling of strain KK8 cells exposed to the filtrate of strain KK22 [KK8(KK22)] or the control KK8 culture without the filtrate. (**a**) Volcano plot showing DEGs: red dots indicate 362 genes significantly up-regulated in KK8(KK22) (log_2_FC>0.5, FDR<0.05), while blue dots represent 379 genes significantly down-regulated (log_2_FC<−0.5, FDR<0.05) compared with the control KK8. (**b**) Predicted functions of the identified DEGs based on COG classification (coloured as in Fig. 1b). Relative abundances (%) of the most abundant or significantly enriched categories are presented: categories [E], [S] and [J] for down-regulated genes, and categories [C], [G], [N] and [J] for up-regulated genes.

To further investigate the response of strain KK8 to exogenous strain KK22 filtrate, transcriptomic profiles were compared between strain KK8 cells incubated with the strain KK22 filtrate [KK8(KK22)] and those incubated without it (control KK8). The RNA-seq reads obtained from these cultures were successfully mapped to the assembled genome of strain KK8, with an average mapping rate of 97.9%. A total of 741 DEGs were identified: 362 genes were significantly up-regulated (log_2_FC>0.5, FDR<0.05) in KK8(KK22), while 379 genes were significantly down-regulated (log_2_FC<−0.5, FDR<0.05) in KK8(KK22) compared with the control KK8 culture (Fig. 4a).

**Fig. 5.**
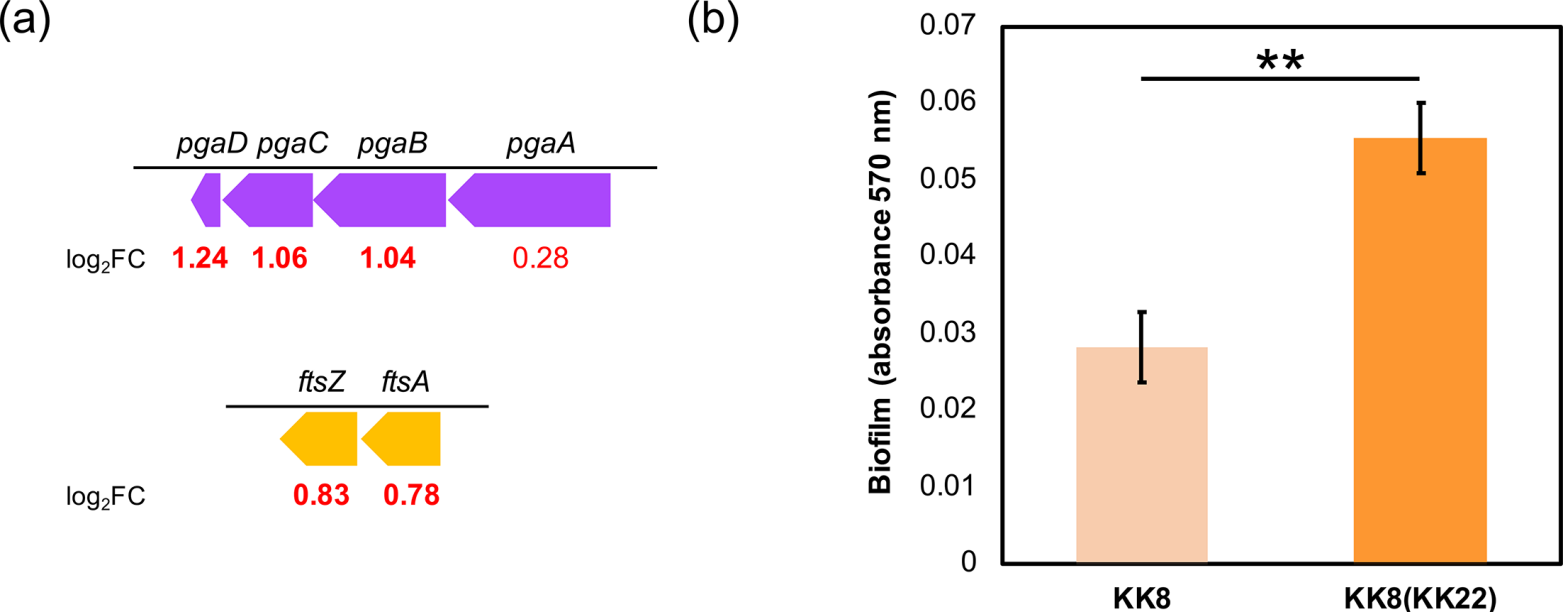
(**a**) Selected functional genes of strain KK8 involved in biofilm formation (*pgaABCD*) or cell division (*ftsA* and *ftsZ*) whose expression was up-regulated in the KK8(KK22) culture, shown with their identified log_2_FC values. (**b**) Comparison of biofilm formation between mature cultures of KK8(KK22) (orange bar) and the control KK8 (light orange bar). **P<0.01 (Welch’s t-test); error bars represent sd (*n*=3).

Among the up-regulated genes, those assigned to the COG functional category ‘[C] Energy production and conversion’ were the most prevalent (53 genes, 14.6% of total up-regulated genes), followed by ‘[E] Amino acid transport and metabolism’ (41 genes, 11.3%). Comparative analysis with the COG category distribution in the whole genome of strain KK8 (Fig. 1b) revealed that genes classified under ‘[N] Cell motility’ – mainly composed of flagellar motor/biosynthesis genes (*mot*, *flh*, *flg* and *fli* genes; Table S5) – were the most enriched among the up-regulated genes (4.39-fold enriched), followed by those under ‘[C]’ (2.32-fold) and ‘[G] Carbohydrate transport and metabolism’ (1.60-fold) (Fig. 4b). The top 10 most significantly up-regulated genes (Table 1) included *pgaD*, encoding a biofilm poly-*β*-1,6-*N*-acetylglucosamine (PGA) synthesis protein (log_2_FC=1.24). Notably, neighbouring genes within the same operon (*pgaABCD*, IMG gene IDs 8120046690–8120046693) encoding PGA synthesis proteins [49] were found significantly up-regulated: *pgaB*, log_2_FC=1.04; *pgaC*, log_2_FC=1.06; although the up-regulation of *pgaA* was not significant (log_2_FC=0.28; Fig. 5a). In addition, genes encoding the cell division proteins FtsA and FtsZ were up-regulated in KK8(KK22) (Fig. 5a), suggesting that these functions may be related to the effects of that strain KK22 filtrate on the growth of strain KK8. The up-regulated genes also included multiple genes involved in the type III secretion system (Table S6).

**Table 1.** Top 10 most up-regulated and down-regulated genes in strain KK8 in the presence of the strain KK22 filtrate

IMG gene ID	Product	COG category	log_2_FC	FDR
**Up-regulated genes**				
8120046610	Xylonate dehydratase	E	1.438	1.35×10^−14^
8120045665	Acetyl-CoA acetyltransferase	I	1.304	5.58×10^−4^
8120047339	Tetratricopeptide (TPR) repeat protein	R	1.299	3.85×10^−14^
8120046810	Haem exporter protein A	O	1.270	6.86×10^−3^
8120045124	(2R)-sulfolactate sulfo-lyase subunit beta	G	1.242	1.47×10^−10^
8120046690	Biofilm PGA synthesis protein PgaD	–	1.237	1.19×10^−4^
8120045159	Vitamin B12 transport system substrate-binding protein	P	1.143	1.60×10^−8^
8120044874	Biotin-dependent carboxylase-like uncharacterized protein	E	1.138	2.45×10^−8^
8120044872	Acetyl-CoA carboxylase, biotin carboxylase subunit	I	1.119	1.82×10-^8^
8120046079	2-methylcitrate dehydratase	G	1.111	1.10×10^−10^
**Down-regulated genes (excluding function-unknown proteins**)				
8120046804	Cytochrome c biogenesis protein CcmG	O	−2.237	4.44×10^−4^
8120046788	Cytochrome c biogenesis protein CcmG	O	−1.697	1.31×10^−2^
8120045044	3-oxoadipate CoA-transferase, beta subunit	I	−1.680	1.48×10^−3^
8120048223	Iron complex transport system ATP-binding protein	P	−1.598	1.37×10^−2^
8120048466	Peptide/nickel transport system permease protein	E	−1.542	7.45×10^−3^
8120048224	Iron complex transport system permease protein	P	−1.490	3.53×10^−3^
8120047456	TonB family protein	–	−1.472	4.69×10^−4^
8120047456	RES domain-containing protein	S	−1.400	9.89×10^−3^
8120045711	P-type Mg^2+^ transporter	P	−1.364	1.43×10^−4^
8120048672	Cardiolipin synthase A/B	I	−1.325	3.34×10^−3^

Functional genes assigned to the category ‘[E] Amino acid transport and metabolism’ were also the most abundant among the down-regulated genes (42 genes, 10.6% of total down-regulated genes; Fig. 4b). Compared with the whole genome, genes under category ‘[S] Function unknown’ and category ‘[J] Translation, ribosomal structure and biogenesis’ were found to be enriched 4.10-fold and 3.63-fold, respectively, among down-regulated genes. Two copies of genes encoding cytochrome c biogenesis protein CcmG (IMG gene IDs 8120046788 and 8120046804), as well as genes involved in the iron complex transport system (IMG gene IDs 8120046788 and 8120046804), were identified as the most down-regulated genes, excluding those of unknown function (Table 1). Other down-regulated genes included those involved in iron uptake (Table S7) and ribosome maturation (i.e. *rimI*, *rimM* and *rimP* genes; Table S8).

### Enhanced biofilm formation of strain KK8 in response to the strain KK22 filtrate

**Fig. 6.**
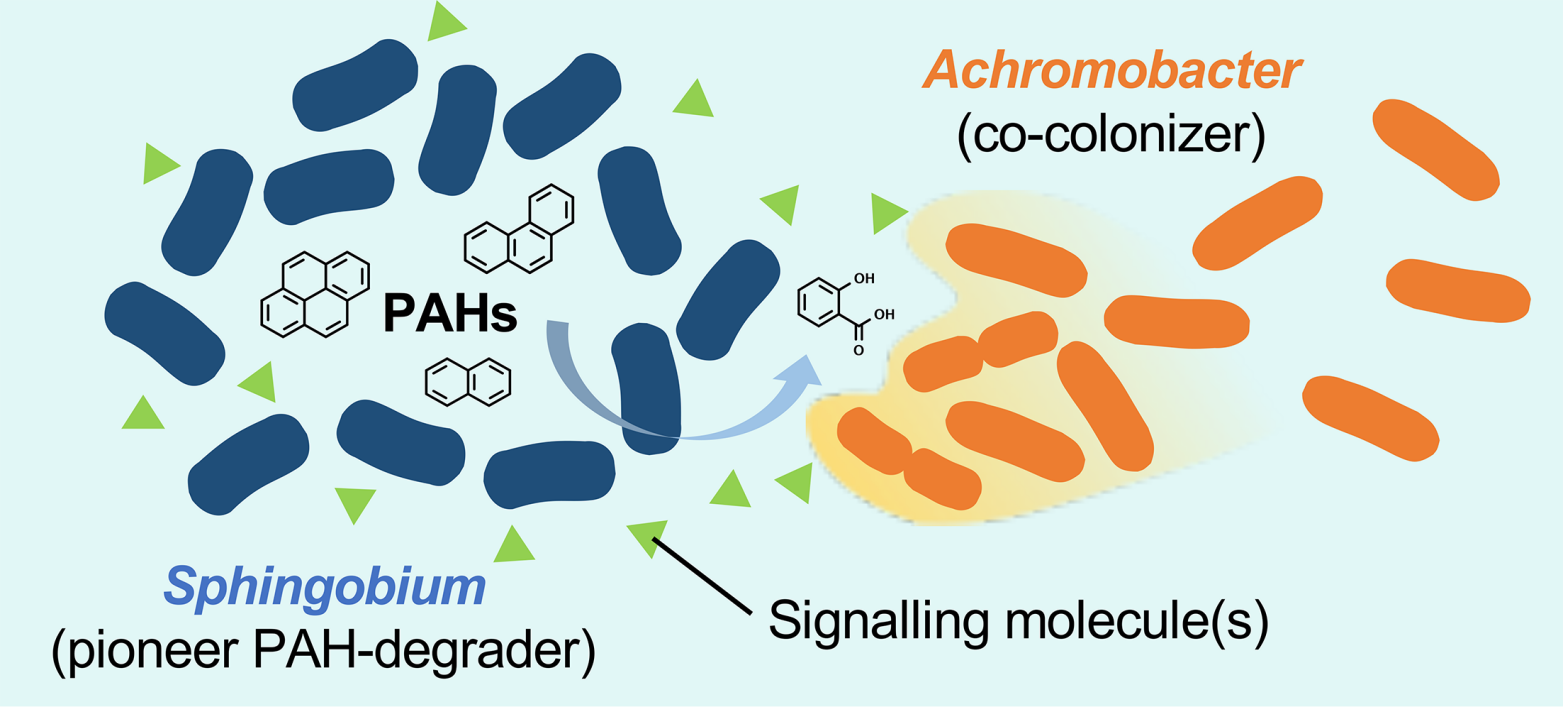
Schematic model summarizing the interactions between a pioneering PAH-degrading *Sphingobium* and the repeatedly documented dominant co-colonizer *Achromobacter* within a PAH-exposed bacterial community. *Sphingobium* initiates the biotransformation of PAHs, while *Achromobacter* grows by utilizing single-ring aromatic compounds provided as PAH biotransformation products. In addition to this metabolic dependency, unidentified signalling molecule(s) released by *Sphingobium* appear to trigger colonization (e.g. cell division and biofilm formation) of *Achromobacter*, likely contributing to their high abundance by outcompeting other bacterial groups.

As transcriptomic analyses suggested that exposure to the strain KK22 filtrate enhances biofilm formation of strain KK8 through up-regulation of *pga* genes, biofilm formation was quantified and compared between cultures of strain KK8 statically incubated with the filtrate [KK8(KK22)] or without it (control KK8), after 25 h of incubation for KK8(KK22) and 30 h for the control KK8. As a result, exposure to the strain KK22 filtrate significantly enhanced biofilm formation in strain KK8, leading to a 1.96-fold increase in biofilm quantity in KK8(KK22) compared with the control KK8 (*P*<0.01; Fig. 5b). This finding suggests that undefined extracellular molecule(s) present in the strain KK22 filtrate triggered biofilm formation in strain KK8 (Fig. 6), via up-regulation of *pga* genes.

## Discussion

Bacteria belonging to the genus *Achromobacter*, which are also well-recognized as opportunistic pathogens infecting persons with cystic fibrosis [50], have been repeatedly identified in aromatic hydrocarbon-enriched bacterial communities; several *Achromobacter* strains have been claimed to be capable of degrading various PAHs, including benzo[*a*]pyrene (*A. xylosoxidans* strain B-2) [51], chrysene (*Achromobacter aegrifaciens* strain S5) [52], pyrene, phenanthrene (*A. xylosoxidans* strains PY4 and LH-1) [53,55], anthracene (*A. xylosoxidans* strain BUK_BTEG6) [56] and fluoranthene (*A. xylosoxidans* strain DN002) [57]. However, none of these previously reported strains have been characterized, through whole-genome sequencing, to possess potential homologues of the well-studied functional marker genes for PAH biotransformation, namely *pah*, *phn* or *nah* genes [58,59]; thus, the presence and conservation of these PAH-degrading enzymes in *Achromobacter* remain open questions. In contrast, regarding functional genes responsible for the biotransformation of single-ring aromatic hydrocarbons, *nagGH* (encoding salicylate 5-hydroxylase) and *nagI* (encoding gentisate 1,2-dioxygenase), which were identified in the genome of strain KK8, are found in reference genomes of *Achromobacter* in public databases without exception (Table S4). Some of these reference genomes additionally contain *catA* (catechol 1,2-dioxygenase; EC 1.13.11.1), *xylE* (catechol 2,3-dioxygenase; EC 1.13.11.2) and *pcaGH* (protocatechuate 3,4-dioxygenase; EC 1.13.11.3) genes, whereas these genes were not found in strain KK8 (Table S4). As in the case of strain KK8, putative homologues of functional genes involved in upstream PAH biodegradation were not found in these reference genomes (Table S4). The ANI between the strain KK8 genome and a representative metagenome-assembled genome (MAG) of *Achromobacter* from the original soil bacterial consortium described in the previous study [11] was 83.56%, and this MAG shared key features with strain KK8, including the presence of *nag* genes and the absence of genes involved in upstream PAH degradation (Table S4). Accordingly, the ability to biodegrade these single-ring aromatic hydrocarbons and to form ecological niches by utilizing these compounds as growth substrates appears to be a well-conserved characteristic among *Achromobacter* species. Previously, salicylic acid, a confirmed growth substrate of strain KK8, was detected and identified as a phenanthrene biotransformation product in cultures of *S. barthaii* strain KK22 grown on phenanthrene [11], suggesting that *Sphingobium* within the consortium provides PAH biotransformation products to *Achromobacter* as growth substrates. In contrast to strain KK8, which possesses *nag* genes responsible for the degradation of salicylic acid via gentisic acid but lacks catechol degradation genes, strain KK22 appeared to degrade phenanthrene through the conversion of salicylic acid to catechol and subsequent catechol degradation, while lacking *nag* genes [9] (Fig. 2). Consistent with this, although both strains KK8 and KK22 are capable of growing on salicylic acid as the sole carbon source, only strain KK8 has previously been characterized as being able to grow on gentisic acid [11], supporting the linkage between genomic data and metabolic functionalities.

The filtrate-exchange experiment revealed that the cell-free filtrate of strain KK22 shortened the growth lag phase of strain KK8 (Fig. 3). Thus, in addition to supplying PAH degradation products for *Achromobacter*, the co-existence of *Sphingobium* within the consortium may facilitate the colonization of *Achromobacter*. Additionally, transcriptomic profiling and subsequent biofilm formation assays of strain KK8 further indicated that exposure of strain KK8 cells to the strain KK22 filtrate enhanced biofilm formation through up-regulation of the *pgaABCD* genes encoding PGA biosynthesis (Fig. 5). The *pgaABCD* gene cluster has been well characterized as being essential for cell-to-cell and cell-to-surface adhesion for biofilm formation [49,60]. Concurrently, the flagellar motility of strain KK8 appeared to be enhanced via up-regulation of flagellar motor/biosynthesis genes (Table S5), which has been reported to be decisive for biofilm formation in *Achromobacter* [61]. The strain KK22 filtrate also appeared to up-regulate genes encoding the cell division proteins FtsZ/FtsA (Fig. 5a), as well as proteins involved in the type III secretion system (Table S6) of strain KK8, thereby likely facilitating the adaptation and replication of *Achromobacter* in the new habitat [62]. On the other hand, genes involved in cytochrome c biogenesis (*ccmG*; Table 1), iron uptake (Table S7) and ribosome maturation (Table S8) were down-regulated in the presence of the strain KK22 filtrate. A previous study reported increased iron uptake and ribosome biogenesis in *Salmonella* during the lag phase [63], suggesting that the lag phase of strain KK8 may have been prolonged in the absence of the strain KK22 filtrate, and cytochrome c appeared to be actively generated in strain KK8 during this extended lag phase.

Taken together, these results suggest that as-yet-unidentified signal molecule(s) produced by *Sphingobium* may specifically promote the co-colonization of *Achromobacter* within the consortium by shortening their growth lag phase and triggering biofilm formation. These effects, in addition to the provision of growth substrates, may have contributed to the high abundance of *Achromobacter* observed in the consortium (Fig. 6). Conversely, the co-occurrence of non-PAH-degrading bacteria may also benefit the PAH degraders by reducing PAH degradation intermediates that exert growth-inhibitory effects on the PAH degraders [64,65]. While phenanthrene, a model low-molecular-weight PAH, has been shown to be completely degraded by strain KK22, exposure to more complex PAHs or hydrocarbon mixtures may require the co-occurrence and cooperation among *Sphingobium* and *Achromobacter* to achieve efficient and complete biodegradation. Previous studies have shown that bacterial quorum-sensing molecules can mediate the development of multispecies mixed biofilms [66,67]. As other possible candidates, exogenous polyamines have been reported to enhance biofilm formation, mediate bacterial host interactions and/or contribute to pathogenesis [68,69]. Consistent with this, genes encoding components of the putative spermidine/putrescine transport system in strain KK8 were found up-regulated in the presence of the strain KK22 filtrate (substrate-binding protein, log_2_FC=0.81; ATP-binding protein, log_2_FC=0.79). Another study reported enhanced growth and biofilm formation through interspecies interactions in a cell-free filtrate-exchanging experiment between marine bacteria, in which branched-chain amino acids were suspected to mediate the interaction [70]. It is therefore possible that similar signalling molecules, or signalling molecule mixtures, released from *Sphingobium* facilitate the co-occurrence of *Achromobacter*, which has been repeatedly documented in PAH-degrading bacterial communities. Further metabolomic investigations of secondary metabolites released by *Sphingobium* may help identify the specific signalling molecule(s) mediating this interspecies interaction, which may be broadly conserved in similar ecosystems. Understanding the mechanisms underlying their co-occurrence shall be valuable for future biotechnological applications of these bacteria.

## Supplementary material

10.1099/mic.0.001712 Supplementary Material 1.

## Abbreviations

ANI: average nucleic acid identity
ARCDs: aromatic ring-cleaving dioxygenases
ARHDs: aromatic ring-hydroxylating dioxygenases
COG: Clusters of Orthologous Groups
DEGs: differentially expressed genes
FDR: false discovery rate
IMG: Integrated Microbial Genomes
log_2_FC: log_2_ fold change
MAG: metagenome-assembled genome
PAHs: polycyclic aromatic hydrocarbons
PGA: poly-*β*-1,6-*N*-acetylglucosamine
SBM: Stanier’s basal medium
