# Supplementary material for: Genomic and transcriptomic insights into Achromobacter–Sphingobium co-colonization within polycyclic aromatic hydrocarbon-exposed bacterial communities
Source: Microbiology (Reading). 2026 May 21;172(5):001712. doi: 10.1099/mic.0.001712 (PMC13193623; doi:10.1099/mic.0.001712)
Supplement: Supplementary Material 1. [file mic-172-01712-s001.pdf]

Supplementary Information

Genomic and transcriptomic insights into *Achromobacter-Sphingobium* co-colonization within polycyclic aromatic hydrocarbon-exposed bacterial communities

Mana Sato, Robert A. Kanaly, and Jiro F. Mori

Graduate School of Nanobioscience, Yokohama City University, Japan

Figure S1. Visual validation of the circular chromosome of strain KK8 using Bandage.

Table S1. Detailed information on PacBio long-read sequencing results.

Table S2. Average nucleic acid identity (ANI) among *A. xylosoxidans* strain KK8 and other *Achromobacter* strains.

Table S3. Functional genes involved in salicylic acid biodegradation in strain KK8 and reference *Achromobacter* strains.

Table S4. Conservation of aromatic hydrocarbon biodegradation proteins in the genomes of strain KK8 and reference *Achromobacter* strains.

Table S5. Expression levels of flagellar motor/biosynthesis genes in the KK8(KK22) culture compared to the control.

Table S6. Expression levels of type III secretion system genes in the KK8(KK22) culture compared to the control.

Table S7. Expression levels of iron uptake genes in the KK8(KK22) culture compared to the control.

Table S8. Expression levels of ribosome maturation genes in the KK8(KK22) culture compared to the control.

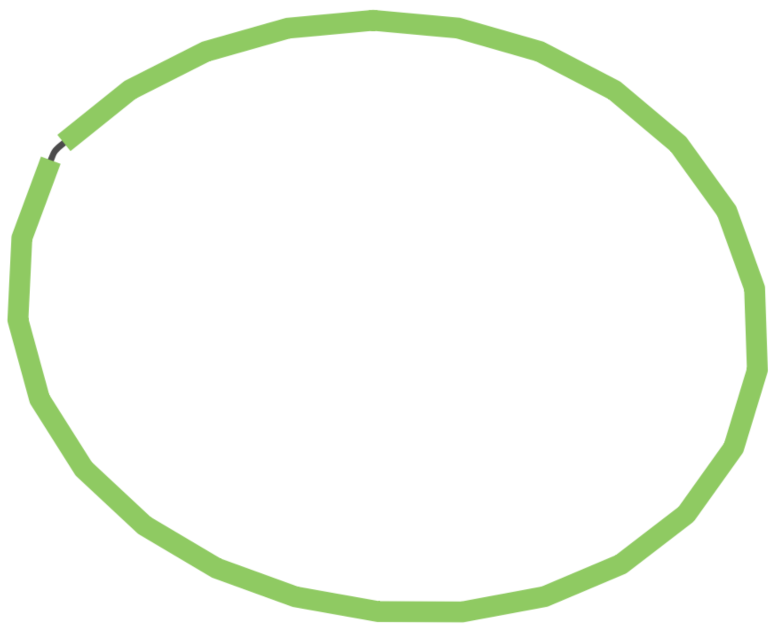

Figure S1. Visual validation of the circular chromosome of strain KK8 using Bandage.

Table S1. Detailed information on PacBio long-read sequencing results.

Number of reads	Average read length (bp)	Total read size (bp)	Assembly total size (bp)	Assembly GC content (%)	Assembly coverage
19,346	12,778	247,209,764	6,394,662	67.8	38.7

Table S2. Average nucleic acid identity (ANI) among *A. xylosoxidans* strain KK8 and other *Achromobacter* strains.

	**KK8**	**SOLR 10**	**NBRC 15126**	**NCTC 10807**	**C54**	**FDAA RGOS _1091**	**NH447 84- 1996**	**MN001**	**AB2**
**A. *xlyosoxidans* KK8**		99.10%	99.01%	99.00%	98.99%	98.95%	98.68%	93.32%	85.21%
**A. *xlyosoxidans* SOLR10**	99.10%		99.31%	99.29%	99.20%	99.00%	98.6%	93.30%	85.22%
**A. *xlyosoxidans* NBRC 15126**	99.01%	99.31%		100%	99.17%	98.86%	98.50%	93.29%	85.22%
**A. *xlyosoxidans* NCTC 10807**	99.00%	99.29%	100%		99.19%	98.83%	98.50%	93.25%	85.19%
**A. *xlyosoxidans* C54**	98.99%	99.20%	99.17%	99.19%		98.97%	98.66%	93.36%	85.29%
**A. *xlyosoxidans* FDAARGOS_1091**	98.95%	99.00%	98.86%	98.82%	98.97%		98.68%	93.26%	85.18%
**A. *xlyosoxidans* NH44784-1996**	98.68%	98.63%	98.50%	98.48%	98.66%	98.68%		93.23%	85.23%
**A. *xlyosoxidans* MN001**	93.32%	93.30%	93.29%	93.25%	93.36%	93.25%	93.23%		85.38%
**A. *insolitus* AB2**	85.21%	85.22%	85.22%	85.19%	85.29%	85.18%	85.21%	85.37%	

Table S3. List of functional genes involved in salicylic acid biodegradation in strain KK8.

IMG gene ID	Product	EC	AA length	AA identity (%) with *A. xylosoxydans* SOLR10	AA identity (%) with *A. insolitus* AB2
8120045646	NagG, salicylate 5-hydroxylase large subunit	1.14.13.172	444	98.4	95.0
8120045645	NagH, salicylate 5-hydroxylase small subunit	1.14.13.172	157	100	92.4
8120045644	NagAb, naphthalene 1,2-dioxygenase ferredoxin component	-	103	100	87.4
8120045648	NagR, DNA-binding transcriptional LysR family regulator	-	300	99.7	91.0
8120045199	NagI, gentisate 1,2-dioxygenase	1.13.11.4	350	99.7	92.9
8120045197	NagL, 3-maleylpyruvate cis-trans-isomerase	5.2.1.4	212	99.1	83.5
8120045198	NagK, fumarylpyruvate hydrolase	3.7.1.20	232	97.8	88.4

Table S4. Conservation of aromatic hydrocarbon biodegradation proteins in the genomes of strain KK8 and reference *Achromobacter* strains.

**Bacterial strains (IMG Genome ID)**	**Proteins (% amino acid identity to strain KK8)**					
	NagGH	NagI	CatA	XylE	PcaGH	NahAcAd,
A.xylosoxidans KK8 (8120043210)	+	+	-	-	-	-
A. xylosoxidans FDAARGOS_1091 (8120369991)	+ (99.0, 99.4)	+ (99.7)	-	-	-	-
A. denitrificans FDAARGOS_786 (8120163383)	+ (93.9, 91.1)	+ (92.6)	-	-	-	-
A. insolitus NCTC13520 (8119224448)	+ (93.6, 92.4)	+ (92.9)	+	+	-	-
A. spanius DSM 23806 (2802429335)	+ (93.3, 91.1)	+ (91.4)	-	-	+	-
A. seleniivolatilans R39 (8129129645)	+ (92.2, 91.7)	+ (92.0)	-	-	+	-
Achromobacter MAG from the consortium	+ (95.4, 92,4)	+ (93.1)	+	-	-	-

Table S5. Expression levels of flagellar motor/biosynthesis genes in the KK8(KK22) culture compared to the control.

IMG gene ID	Product	COG category	Log_2 fold change	FDR
8120044321	MotB, flagellar motor protein	N	0.625	7.76×10^{-5}
8120047047	FlhF, flagellar biosynthesis protein	N	0.603	2.11×10^{-5}
8120047053	FlgD, flagellar basal-body rod modification protein	N	0.585	4.97×10^{-6}
8120047054	FlgE, flagellar hook protein	N	0.790	4.31×10^{-10}
8120047055	FlgF, flagellar basal-body rod protein	N	0.629	5.93×10^{-7}
8120047056	FlgG, flagellar basal-body rod protein	N	0.716	2.97×10^{-9}
8120047057	FlgH, flagellar L-ring protein	N	0.641	5.16×10^{-5}
8120047070	FliO/FliZ, flagellar protein	N	0.650	4.63×10^{-2}
8120047071	FliN, flagellar motor switch protein	N	0.770	4.02×10^{-4}
8120047072	FliM, flagellar motor switch protein	N	0.662	1.29×10^{-5}
8120047073	FliL, flagellar protein	N	0.539	2.41×10^{-4}
8120047074	FliK, flagellar hook-length control protein	N	0.977	9.28×10^{-11}
8120047075	FliJ, flagellar protein	N	1.099	6.95×10^{-11}
8120047077	FliH, flagellar assembly protein	N	0.634	7.62×10^{-5}

Table S6. Expression levels of type III secretion system genes in the KK8(KK22) culture compared to the control.

IMG gene ID	Product	COG category	Log_2 fold change	FDR
8120045367	type III secretion system low calcium response chaperone LcrH/SycD	R	0.606	2.19×10^{-5}
8120045370	type III secretion system low calcium response chaperone LcrH/SycD	R	0.659	3.12×10^{-5}
8120045376	ATP synthase in type III secretion protein N	N	0.723	1.97×10^{-3}
8120045377	type III secretion protein O	C	1.061	8.86×10^{-3}
8120045379	type III secretion protein Q	N	0.658	2.39×10^{-2}
8120045380	type III secretion protein R	U	0.747	4.92×10^{-2}
8120045381	type III secretion protein S	U	1.091	4.65×10^{-2}
8120045385	type III secretion protein C	U	0.683	9.39×10^{-5}

Table S7. Expression levels of iron uptake genes in the KK8(KK22) culture compared to the control.

IMG gene ID	Product	COG category	Log_2 fold change	FDR
8120043558	iron complex transport system ATP-binding protein	P	-0.033	1.00
8120043559	iron complex transport system permease protein	P	-0.115	8.94×10^{-1}
8120043560	iron complex transport system substrate-binding protein	P	-0.351	4.54×10^{-1}
8120045818	iron complex outermembrane receptor protein	P	-0.410	3.16×10^{-1}
8120045819	iron complex transport system substrate-binding protein	P	-0.523	3.95×10^{-1}
8120045820	iron complex transport system permease protein	P	-0.646	3.67×10^{-1}
8120045823	bacterial IrtB/YbtQ, ATP-binding cassette, subfamily B	V	-0.376	4.51×10^{-1}
8120045824	bacterial IrtA/YbtP, ATP-binding cassette, subfamily B	V	-0.154	7.33×10^{-1}

Table S8. Expression levels of ribosome maturation genes in the KK8(KK22) culture compared to the control.

IMG gene ID	Product	COG category	Log_2 fold change	FDR
8120043224	large subunit ribosomal protein L28	J	-0.749	2.09×10^{-4}
8120043225	large subunit ribosomal protein L33	J	-0.621	1.31×10^{-2}
8120044268	large subunit ribosomal protein L21	J	-0.596	1.70×10^{-3}
8120044269	large subunit ribosomal protein L27	J	-0.538	3.26×10^{-3}
8120044555	large subunit ribosomal protein L24	J	-0.637	9.75×10^{-4}
8120044556	large subunit ribosomal protein L14	J	-0.885	1.62×10^{-5}
8120044583	small subunit ribosomal protein S12	J	-0.657	1.36×10^{-3}
8120044649	large subunit ribosomal protein L34	J	-0.677	2.63×10^{-3}
8120045836	small subunit ribosomal protein S15	J	-0.563	1.14×10^{-3}
8120046002	large subunit ribosomal protein L32	J	-0.524	1.30×10^{-3}
8120046216	large subunit ribosomal protein L31	J	-0.725	3.45×10^{-5}
8120046530	small subunit ribosomal protein S16	J	-0.502	1.42×10^{-3}
8120046555	small subunit ribosomal protein S21	J	-0.591	2.97×10^{-4}
8120046642	large subunit ribosomal protein L35	J	-0.567	2.55×10^{-3}
8120048378	small subunit ribosomal protein S20	J	-0.696	2.07×10^{-5}
8120046236	ribosome maturation factor RimP	J	-1.197	3.94×10^{-11}
8120046531	16S rRNA processing protein RimM	J	-0.569	5.61×10^{-4}
